# Adherence to the Alternate Mediterranean Diet and Multidimensional Sleep Health Among US Adults from the 2017–2018 National Health and Nutrition Examination Survey: Exploring Racial/Ethnic Disparities

**DOI:** 10.1016/j.jand.2026.156300

**Published:** 2026-01-27

**Authors:** Astrid N. Zamora, Velarie Y. Ansu-Baidoo, Erica C. Jansen

**Affiliations:** Department of Epidemiology and Population Health, School of Medicine, Stanford University, Stanford, CA.; Department of Psychiatry, Center for Translational Sleep and Circadian Sciences, School of Medicine, University of Miami Miller, Miami, FL.; Assistant Professor, Department of Nutritional Sciences, School of Public Health, University of Michigan, Ann Arbor, MI, and a research assistant professor, Department of Neurology, University of Michigan Medical School, Ann Arbor, MI.

**Keywords:** Alternate Mediterranean Diet, Dietary Patterns, NHANES, Race and ethnicity, Sleep

## Abstract

**Background:**

Adherence to the Mediterranean diet has been linked to better sleep health. However, the relationship between adherence to the alternate Mediterranean diet (aMED) and specific sleep health dimensions remains understudied, particularly among racial/ethnic minority adult populations in the United States (US).

**Objective:**

The objective was to examine associations between aMED adherence and multiple dimensions of sleep health in US adults and assess differences by race/ethnicity.

**Design:**

A cross-sectional analysis using data from the 2017–2018 National Health and Nutrition Examination Survey (NHANES), a nationally representative survey of the noninstitutionalized US population, was conducted. Dietary intake was assessed via two 24-hour recalls to calculate a modified aMED score, and sleep health was measured using self-reported dimensions summarized into a composite score.

**Participants/Setting:**

The analytic study sample included 3005 adults (aged 18 years and older) from the 2017–2018 NHANES cycle. Analyses were survey-weighted to yield estimates representative of US adults who completed two 24-hour dietary recalls.

**Main Outcome Measures:**

Sleep health was assessed using 5 self-reported dimensions (ie, regularity, timing, duration, satisfaction, and alertness) summarized into a composite multidimensional sleep health score, with a score ≥3 indicating overall “good” sleep health.

**Statistical Analyses Performed:**

Survey-weighted logistic regression models were used to examine the associations between aMED adherence and sleep health dimensions as well as the overall multidimensional sleep health score, while adjusting for confounding variables. All models incorporated NHANES strata, clusters, and the 2-day dietary recall weight (WTDR2D) to account for the complex survey design and obtain nationally representative estimates. Effect modification by race/ethnicity was assessed using both interaction terms and stratified models.

**Results:**

In survey-weighted analyses, the mean (SE) age was 47.1 (0.6) years; 51.3% were male. The weighted mean aMED score was 4.0, and 71% of adults were classified as having good sleep health. Higher aMED scores were significantly associated with greater odds of achieving recommended sleep duration (odds ratio [OR] per 1-point increase 1.1; 95% CI, 1.0 to 1.2; *P* = .03). Stratified analyses revealed that moderate/high aMED adherence was significantly associated with a greater odds of achieving recommended sleep duration among racial/ethnic minority adults (OR, 1.3; 95%, CI 1.1 to 1.7; *P* = .01), but not among non-Hispanic White adults (OR 1.1; 95% CI, 0.7 to 1.9; *P* = .68). No significant associations were observed for other sleep domains or the overall multidimensional sleep health score.

**Conclusions:**

Higher aMED adherence was associated with greater odds of achieving recommended sleep duration, with this association observed among racial/ethnic minority adults, but not among non-Hispanic White adults. Given the cross-sectional design, temporality cannot be established. Longitudinal studies incorporating objective sleep measures are needed to further evaluate these associations.

Sleep disturbances, including insufficient sleep duration and poor sleep quality, are significant public health concerns in the United States (US), affecting millions of adults annually.^1^

Insufficient sleep duration, defined as habitually sleeping less than 7 hours per night for adults aged 18 years and older,^2^ has been linked to increased cardiometabolic risk.^3,4^ Moreover, poor sleep quality, characterized by difficulty falling asleep, frequent awakenings, and nonrestorative sleep, contributes to daytime fatigue, reduced productivity, and mental health issues like anxiety and depression.^5,6^ With one-third of US adults reporting insufficient sleep duration,^7^ there is an urgent need to better understand the factors contributing to insufficient sleep.

Several factors contribute to sleep disturbances in adulthood. Behavioral factors such as sedentary behavior,^8^ chronic stress,^9^ and poor sleep hygiene^10,11^ can negatively impact sleep. Psychosocial stressors, including work-related pressures,^12–14^ socioeconomic challenges,^15^ and family responsibilities^16^ also play a significant role. Environmental factors such as noise,^17^ light pollution,^18^ toxicants,^19,20^ and irregular work schedules^21^ further complicate sleep health. In addition, biological factors such as age-related changes^22,23^ and chronic health conditions^24,25^ also contribute to the complexity of sleep disturbances.

Although behavioral and environmental factors are well-established contributors to sleep disturbances, a growing body of research suggests that dietary intake may also play an important role.^26,27^ Much of the existing literature has focused on specific nutrients or food items,^28–31^ including fruits and vegetables.^32–35^ However, there is growing interest in dietary patterns, for example, the Mediterranean diet, which is rich in fruits, vegetables, whole grains, healthy fats, and fish.^36^ The Mediterranean diet has been linked to various health benefits, including improved sleep quality.^37–40^

Beyond epidemiologic associations, several plausible pathways may help explain a connection between the Mediterranean diet and sleep health. This dietary pattern emphasizes nutrient-dense foods that support circadian regulation and neuronal function,^41,42^ while its anti-inflammatory properties may mitigate biological processes linked to poor sleep.^41,42^

Despite this growing evidence base, most prior studies have focused on isolated sleep health measures in relation to adherence to the Mediterranean diet, rather than exploring relationships with multidimensional sleep health measures. For instance, most research has relied on single-dimensional sleep measures, such as sleep duration,^37,39^ ignoring the broader aspects of sleep health, including timing, regularity, satisfaction, and alertness.^43^ Adopting a multidimensional sleep health (MDSH) framework, introduced by Buysse,^43,44^ recognizes that sleep dimensions are distinct yet interrelated. This framework emphasizes positive sleep habits that promote disease-free survival and healthy aging, offering actionable targets for improving sleep health.^43,44^ An MDSH approach can be applied to both individual-level and population-level strategies to promote cardiovascular and cardiometabolic health, providing a more comprehensive understanding of how dietary patterns may influence sleep health,^43,44^

The US is a diverse nation, with distinct cultural, socioeconomic, and health factors influencing sleep patterns.^45–47^ Racial/ethnic minority groups in the US, including Asian and Pacific Islander, African American, Latino/a and Hispanic, Native American, and multi-race populations, generally experience poorer sleep health compared with non-Hispanic (NH) White adult populations.^48,49^ However, to the best of our knowledge, little research has explored how these sleep health disparities intersect with dietary factors, particularly adherence to the Mediterranean diet, which may have varying impacts across these groups.

This cross-sectional study aims to explore associations between adherence to the alternate Mediterranean diet (aMED) pattern and multidimensional sleep health measures using a nationally representative sample of US adults. The aims included: (1) to examine the relationship between aMED adherence and sleep across 5 dimensions of sleep health, including regularity, timing, duration, satisfaction, and alertness; and (2) to investigate how race/ethnicity (NH White adults vs racial/ethnic minority adults) interact with adherence to the aMED diet (low vs moderate/high) and its association with the odds of good sleep health, both overall and for each sleep measure. The hypothesis was that higher adherence to aMED would be associated with greater odds of achieving good sleep health, and that this association would differ by race/ethnicity.

## METHODS

### Study Design and Population

This cross-sectional study used data from the 2017–2018 National Health and Nutrition Examination Survey (NHANES) cycle, a nationally representative survey conducted by the National Center for Health Statistics that assesses the health and nutritional status of the US population.^50^ The NHANES survey combines interviews and physical examinations to collect data from a complex, multistage probability sample.^51^

**Figure 1.**
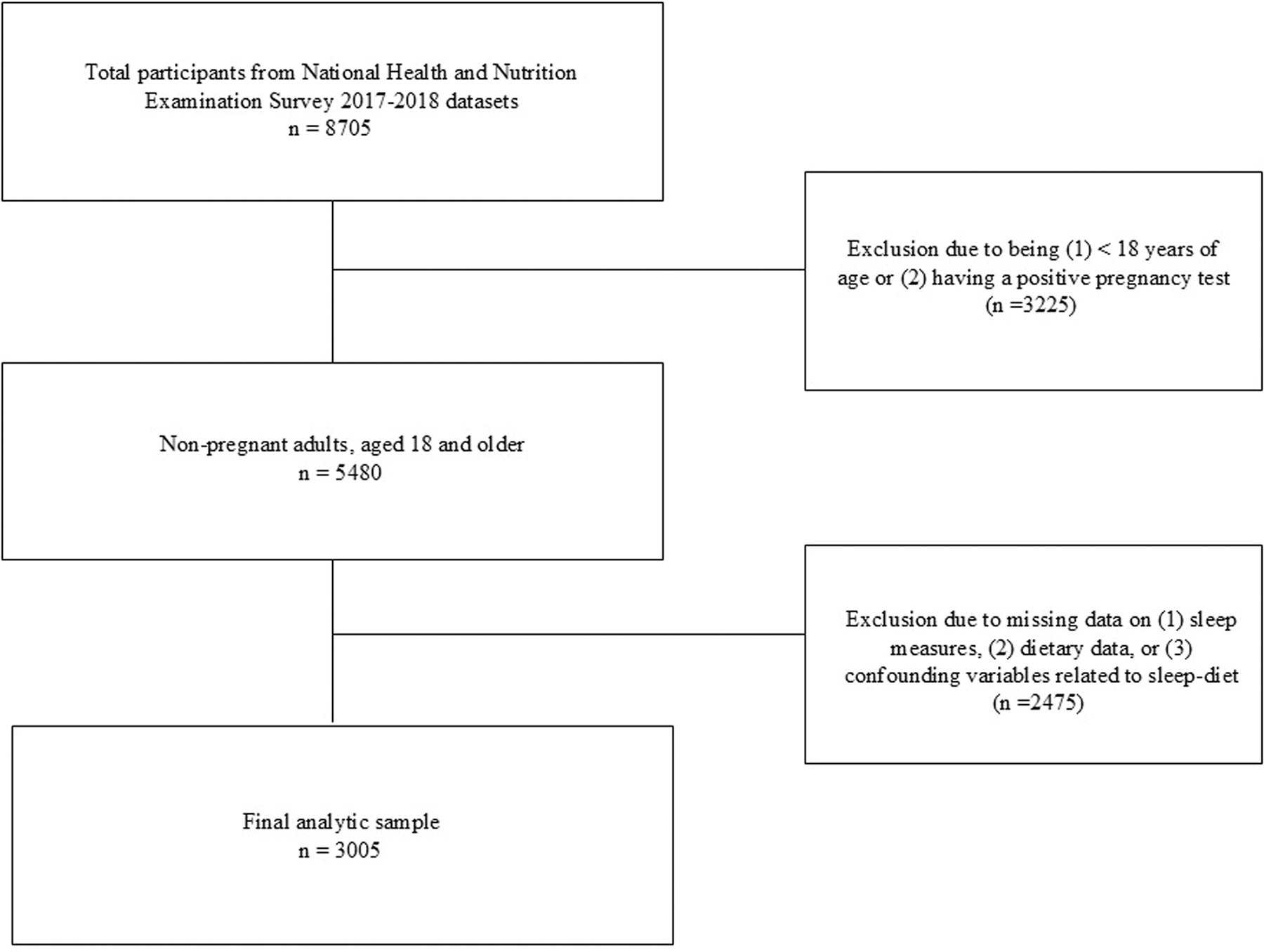
Flow diagram illustrating participant inclusion and exclusion for the analytic sample derived from the 2017–2018 National Health and Nutrition Examination Survey (NHANES) cycle. A total of 8705 participants were initially assessed; exclusions included individuals younger than age 18 years or with a positive pregnancy test (n = 3225), and participants missing data on sleep measures, dietary data, or confounding variables related to sleep—diet associations (n = 2475). The final analytic sample comprised 3005 non-pregnant adults 18 years and older.

Participants in the current investigation were adults aged 18 years and older with complete data for all variables of interest from the 2017–2018 NHANES cycle. Excluded individuals were those who (1) were younger than 18 years, (2) were pregnant, (3) had missing data on any of the sleep health measures of interest, (4) did not have 2 complete and valid 24-hour dietary recalls or the associated 2-day dietary weights (WTDR2D), and (5) were missing data on any covariates identified as potential confounders. The inclusion criteria, therefore, restricted the sample to non-pregnant adults with complete data on 2 dietary recalls, sleep measures, and all potential confounding covariates. The final eligible sample comprised 3005 participants. See Figure 1 for the study flow diagram.

NHANES received approval from the Centers for Disease Control and Prevention and National Center for Health Statistics Ethics Review Board, and all participants provided written informed consent at the time of data collection. As our investigation used publicly available and deidentified NHANES data, it was deemed exempt from human-subject research procedures by the Stanford University Institutional Review Board.

### Assessment of the aMED Score

NHANES dietary data were collected using the US Department of Agriculture (USDA) Automated Multiple-Pass Method,^52^ a structured and validated approach designed to reduce recall bias and misreporting. The first 24-hour dietary recall was administered in person at an NHANES mobile examination center, and the second recall was conducted via telephone approximately 3 to 10 days later.^53^ Reported foods and beverages were coded using the USDA Food and Nutrient Database for Dietary Studies,^54^ which provides the nutrient composition values used in NHANES analyses. To reduce within-person day-to-day variability, only participants with 2 valid dietary recalls and the associated 2-day dietary weights (WTDR2D) were included. By incorporating both recalls, the potential for random error and day-to-day variability was reduced.^55^ NHANES does not guarantee that the 2 dietary recalls represent both a weekday and a weekend day; therefore, dietary intake estimates may not fully capture potential weekend—weekday variability in eating patterns. Although self-reported intake is subject to misreporting and recall limitations,^56^ NHANES’ rigorous interviewer training and quality assurance procedures help mitigate these biases. No imputations were performed for missing dietary data.

The aMED score was calculated using the method developed by Fung and colleagues^57^ and applied to the NHANES dietary data. The aMED components included vegetables, fruits, whole grains, nuts, legumes, fish and seafood, red and processed meat, alcohol intake, and the ratio of monounsaturated to saturated fats (MUFA/SFA). Each component was quantified using the appropriate NHANES variables, for example, total cups per day for vegetables and fruits; ounces per day for whole grains, nuts, legumes, and fish; and grams per day for alcohol. The MUFA/SFA ratio was calculated from the mean fat intake values across both recalls.

To account for sex differences in dietary intake, sex-specific medians were calculated for each dietary component. Beneficial components, vegetables, fruits, whole grains, nuts, legumes, fish, and the MUFA/SFA ratio, were assigned a score of 1 if the participant’s intake was greater than the sex-specific median. The detrimental component, red and processed meat, was scored as 1 if intake was equal to or less than the median. Alcohol intake was scored based on sex-specific thresholds: men received 1 point for moderate intake (10–50 g/d), and women received 1 point for moderate intake (5–25 g/d).

The total aMED score ranged from 0 to 9 points, with higher scores indicating greater adherence to the aMED. For analysis, participants were classified into 1 of 2 groups: those with aMED score ≥4 were categorized as having moderate/high adherence, and those with a score <4 were categorized as having low adherence. Dietary component values were derived from the mean of two 24-hour dietary recalls to reduce within-person day-to-day variation. Although this approach provides a more reliable estimate of intake than a single day, it does not constitute a formal measure of usual intake, which would require statistical modeling approaches, such as the National Cancer Institute’s method.^58^

### Assessment of Sleep Health Measures

Sleep health measures were estimated from participants self-reporting their usual sleep. The following 5 individual sleep health domains were assessed:timing, regularity, duration, satisfaction, and alertness.

**Figure 2.**
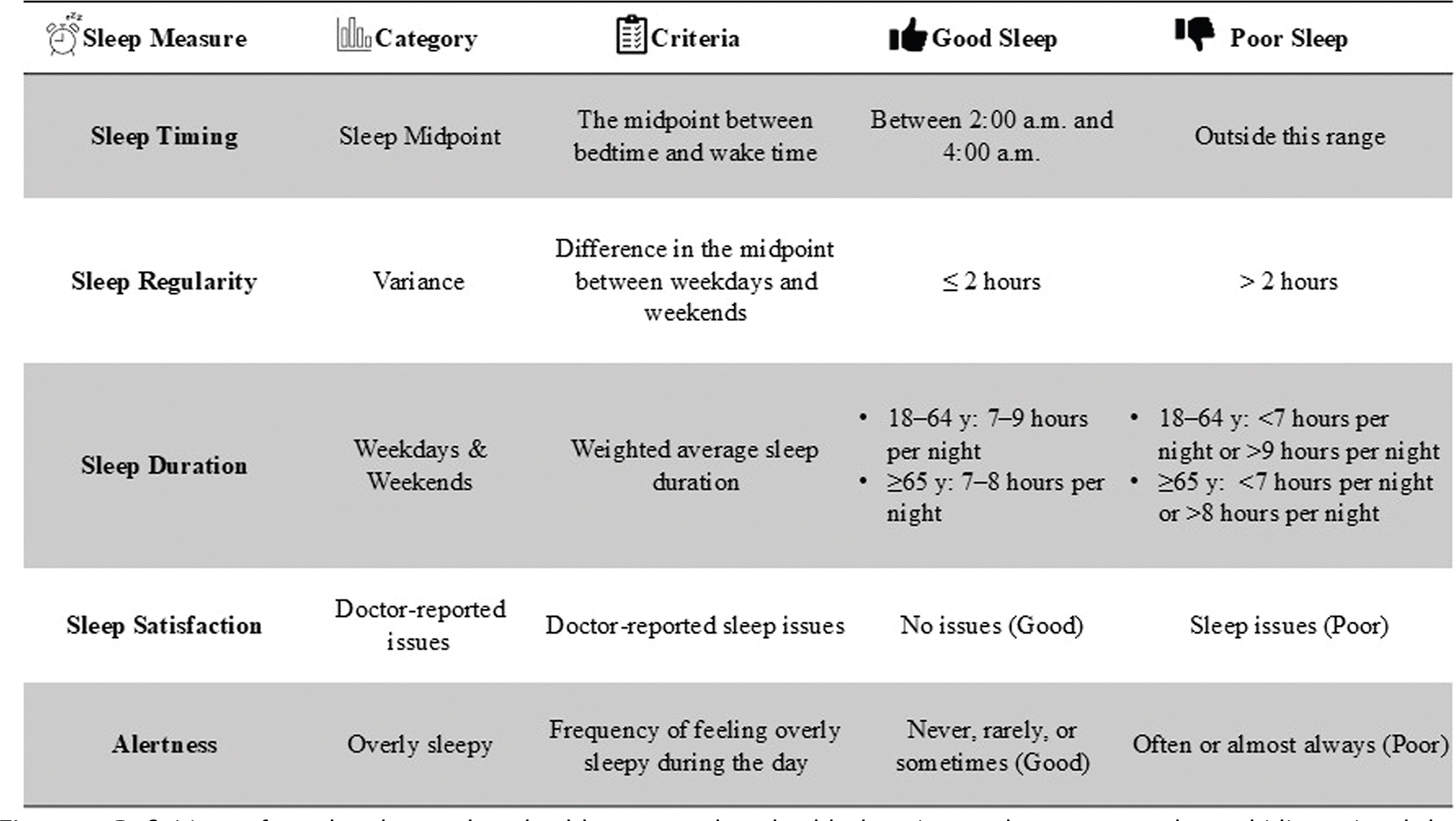
Definitions of good and poor sleep health across 5 sleep health domains used to construct the multidimensional sleep health (MSDH) score. Sleep health domains and criteria are adapted from previously published frameworks^43,44^ and National Sleep Foundation guidelines.^59^

The MDSH score was then calculated by summing the number of “good” indicators across the 5 sleep health domains (range, 0–5, with higher scores indicating better sleep health). For analytic purposes, our study included a binary classification: scores ≥3 were classified as “good” sleep health and scores <3 were classified as “poor” sleep health. Figure 2 summarizes the scoring criteria used to categorize each individual sleep health domain and the composite MDSH score.

Usual sleep and wake times on both weekdays and weekend days were assessed using the Sleep Disorders Questionnaire, which was adapted from the Munich ChronoType Questionnaire.^60^ The Sleep Disorders Questionnaire has undergone psychometric evaluation within NHANES and other population-based studies and is considered a validated measure of habitual sleep patterns.^61^

Sleep timing was assessed by calculating the midpoint between reported bedtime and wake time. When wake time occurred earlier than bedtime (indicating that the sleep period extended past midnight), 24 hours were added to the wake time to ensure the interval was represented correctly. Separate midpoints were calculated for weekdays and weekends, and a mean sleep midpoint was then computed as a weighted mean (5 weekdays and 2 weekend days). Participants were categorized into 1 of the following 2 groups: those with good sleep timing (mean midpoint between 2:00 AM and 4:00 AM) or those with poor sleep timing (mean midpoint outside this range), as defined previously.^43,62^

Sleep regularity was assessed by calculating the difference between the weekday and weekend sleep midpoints. Sleep regularity was classified as good if the absolute difference between the 2 midpoints was ≤2 hours, and poor if the difference was >2 hours. This measure reflects consistency in sleep timing between workdays and weekends, as defined previously.^62^

Sleep duration was calculated as wake time minus reported bedtime for both weekdays and weekends. Total hours slept on weekdays and weekends were then used to calculate average sleep duration (weighted as 5 weekdays and 2 weekend days). Sleep duration was categorized as good or poor according to the National Sleep Foundation guidelines.^59^ For adults aged 18 to 64 years, good sleep duration was defined as 7 to 9 hours per night; for adults aged 65 years and older, it was defined as 7 to 8 hours per night. Any duration outside these ranges was considered poor sleep duration.^59^

Sleep satisfaction was evaluated by asking whether a physician had ever disgnosed sleep troubles. As defined previously,^62^ participants were classified as having good sleep satisfaction if they reported no physician-diagnosed sleep issues, and poor sleep satisfaction if they reported having sleep issues. This pragmatic proxy measure has precedent in prior NHANES analyses.^63^ However, it may not capture the full breadth of subjective sleep satisfaction.

Alertness was assessed by the frequency with which participants reported feeling overly sleepy during the day. As defined previously,^62^ participants who reported feeling overly sleepy never, rarely, or sometimes were classified as having good alertness, whereas those who reported feeling overly sleepy often or almost always were classified as having poor alertness.

These classifications and cutoffs are consistent with those used in prior epidemiologic studies^62^ and align with the MDSH framework proposed by Buysse.^43^

### Sociodemographic and Behavioral Characteristics

Demographic characteristics were obtained from the NHANES demographic file. Age (in years) and poverty-to-income ratio (PIR) were included as continuous variables, with PIR <1.0 indicating income below the federal poverty threshold.^64^ The term “sex” is used in place of “gender,” which is consistent with NHANES coding. Race/ethnicity (defined here as a social construct) were dichotomized into NH White versus all other racial/ethnic minority groups, including Latino/a, NH Black, NH Asian, other, or multiracial, to preserve a sufficient sample size and maintain statistical power consistent with prior approaches when subgroup sample sizes are limited.^65,66^ Marital status was categorized as married, widowed, divorced, or never married. Educational attainment was grouped into <9 years, 9 to 11 years, high school graduate (12 years), and some college or more. Smoking status was classified as never, former, or current smoker, consistent with prior population-based studies.^67^ Although NHANES collects more detailed smoking data, collapsing it into 3 categories allowed us to preserve a sufficient sample size, reduce instability in estimates, and maintain comparability with prior epidemiologic approaches.^67^ Food insecurity was assessed using the 18-item USDA Household Food Security Scale and categorized into 4 levels: full, marginal, low, and very low food security, in accordance with USDA guidelines. Scoring follows the USDA protocol,^68^ which classifies households with children using child-specific items and cutpoints and households without children using adult-only cutpoints. Body mass index (BMI) was calculated as weight in kilograms divided by height in meters squared.

Other behavioral measures included sedentary behavior, moderate-to-vigorous physical activity (MVPA), and daily energy intake. Time spent in sedentary behavior and MVPA was assessed using NHANES physical activity questionnaires, and daily energy intake was derived from 24-hour dietary recalls. Sedentary behavior was assessed based on self-report in response to the following: “The following question is about sitting at work, at home, getting to and from places, or with friends, including time spent sitting at a desk, traveling in a car or bus, reading, playing cards, watching television, or using a computer. Do not include time spent sleeping. How much time do you usually spend sitting on a typical day?” Sedentary behavior was operationalized as a continuous variable, reflecting the number of minutes per day, in all analyses. The total minutes per week of MVPA were estimated using the Global Physical Activity Questionnaire, which includes questions on daily, leisure-time, and sedentary activities. The Global Physical Activity Questionnaire has been validated and found to be a reliable tool for physical activity monitoring in various studies, including NHANES.^69,70^ MVPA minutes per week were computed by summing minutes from occupational, transportation, and leisure-time activities, following established methods.^71^ The mean daily caffeine intake (in milligrams) was derived from the 24-hour dietary recall files (ie, DR1TCAFF and DR2TCAFF) for days 1 and 2 and calculated as the mean of the 2 recalls. Lastly, total dietary energy intake was adjusted for in the models. This approach aligns with methodological recommendations by Willett and colleagues^72^ regarding energy adjustment in nutritional epidemiology, recognizing that total energy intake can confound associations between dietary exposures, such as aMED adherence, and health outcomes, including sleep.

### Statistical Analysis

Descriptive statistics were first computed for the study variables, including the aMED score, sleep health measures, and covariates. Differences in aMED adherence (low vs moderate/high) according to participant characteristics were examined. In accordance with NHANES statistical recommendations,^73^ means and proportions were calculated using PROC SURVEYMEANS and PROC SURVEYFREQ procedures in SAS software, version 9.4.^74^ Due to the complex nature of the survey sample, the Rao-Scott χ^2^ test for categorical variables and the weighted *t* test for continuous variables were used to assess differences. Continuous variables are presented as weighted means and SEs, and categorical variables are presented as unweighted sample sizes and weighted percentages.

For regression analyses, associations were examined between a 1-point increase in the aMED score and the odds of overall good sleep health (binary MDSH outcome) and the odds of good sleep for each individual sleep health measure. Weighted logistic regression models were applied. Three models were developed to examine the association between aMED and sleep outcomes. Model 1 quantified crude associations without covariate adjustment. Model 2 was adjusted for age, sex, race/ethnicity, and total dietary energy intake, which were considered fundamental confounders. Model 3 further adjusted for additional socioeconomic and behavioral factors (ie, total mean caffeine intake, smoking history, education, household food security level, PIR, and BMI), which may influence both diet and sleep. This stepwise modeling strategy was used to illustrate how associations changed as adjustments progressed. Covariates were selected *a priori* based on evidence of associations between aMED and sleep outcomes in prior studies.^37,39,40^ For all regression models, the odds ratios (OR) and corresponding 95% confidence intervals (CIs) are reported.

Interaction terms were used to assess whether the association between adherence to the aMED and sleep health measures differed by race/ethnicity. Specifically, we tested statistical interaction between aMED adherence and race and ethnicity (categorized as NH White vs racial/ethnic minority groups) using the Type 3 Analysis of Effects from the PROC SURVEYLOGISTIC procedure in SAS software, Version 9.4.^74^ This test evaluates the joint significance of the interaction terms. The binary race/ethnicity categorization was informed by prior literature,^75,76^ which often combines racial/ethnic minority groups for statistical power and interpretability. Given the relatively small sample size, disaggregating minority subgroups would have resulted in insufficient power.

In addition to interaction testing, we included post hoc stratified analyses by race and ethnicity to clarify the interpretation of the diet—sleep associations. These analyses estimated associations between aMED adherence and each sleep outcome separately within NH White and racial/ethnic minority adults, using the same covariate adjustment strategy described above. Stratified models were used because interaction terms can be challenging to interpret when using a single reference group. In contrast, stratification provides more direct within-group estimates of association.

All analyses were performed using SAS, version 9.4,^74^ and statistical significance was determined at the .05 level. Because NHANES uses a complex multistage probability sampling design, all analyses incorporated survey strata (SDMVSTRA), primary sampling units (SDMVPSU), and the 2-day dietary recall weight (WTDR2D). This approach yields estimates that are representative of US adults who completed two 24-hour dietary recalls. All analyses were survey-weighted.

## RESULTS

### Participant Characteristics

The mean (SE) age of the sample was 47.1 (0.6) years; 51.3% of the participants were male, and 64.2% identified as NH White. Overall, 59.6% of participants had moderate/high adherence to the aMED diet (score ≥4), with a mean (SE) aMED score of 4.0 (0.09). In addition, 70.8% reported good overall sleep based on the MDSH score.

Several sociodemographic and behavioral characteristics differed by adherence to aMED (Table 1). Compared with the low adherence group, individuals with moderate/high adherence were older (*P* = .001), less likely to be current smokers (*P* < .0001), and more likely to have >12 years of education (*P* < .0001). Moreover, race and ethnicity distributions differed significantly by aMED adherence category (*P* < .0001). Group differences were also observed for household food security (*P* < .0001), PIR (*P* < .0001), and BMI (*P* = .0090). Regarding lifestyle factors, those with moderate/high adherence reported greater total energy intake (*P* < .0001), lower levels of MVPA (*P* = .002), more sedentary behavior (*P* = .045), and lower mean caffeine intake (*P* = .02).

### Aim 1 Findings

The association between continuous aMED scores and the odds of achieving outcomes for the MDSH (binary) and each individual sleep domain (binary) was examined (Table 2). In the fully adjusted model (model 3), a statistically significant association was observed between higher aMED scores and good sleep duration, with each 1-point increase in the aMED score associated with a 10% increase in the odds of achieving good sleep duration (OR 1.1; 95% CI, 1.0 to 1.2; *P* = .03).

No other sleep health outcomes were significantly associated with aMED scores in the fully adjusted model. When examining MDSH as a binary outcome, aMED scores were not associated with achieving overall good sleep health (OR 1.0; 95% CI, 0.9 to 1.2; *P* = .32), and all other sleep health domains, including regularity, satisfaction, alertness, and timing, showed nonsignificant associations as well.

### Aim 2 Findings

Next, we examined the interactions between aMED adherence and race/ethnicity across MDSH and individual sleep domains using the modeling framework described above, with racial/ethnic minority adults with low aMED adherence serving as the reference group (Table 3).

### MDSH

No statistically significant interaction between race/ethnicity and aMED adherence was observed for the MDSH (model 3: *P*_interaction_ = .57). ORs for both NH White adults and racial/ethnic minority adults with moderate/high adherence were close to the null and not statistically significant.

### Sleep Health Domains

A statistically significant interaction was observed for good sleep duration (model 3: *P*_interaction_ =.04). However, no significant interactions were observed for good sleep regularity (*P*_interaction_ = .28), good sleep satisfaction (*P*_interaction_ = .26), good sleep alertness (*P*_interaction_ = .64), or good sleep timing (*P*_interaction_ = .43). ORs across groups were generally close to the null.

### Post Hoc Stratified Analyses by Race/Ethnicity

A post hoc stratified analysis was conducted to aid interpretation of the statistically significant interaction for sleep duration (Table 4). A significant association was observed for sleep duration among racial/ethnic minority adults, with moderate/high adherence to the aMED associated with greater odds of achieving the recommended (good) sleep duration (OR 1.3; 95% CI, 1.1 to 1.7; *P* = .01). In contrast, no significant association was observed among NH White adults (OR 1.1; 95% CI, 0.7 to 1.9; *P* = .68). For MDSH and other individual sleep health domains, no statistically significant associations were detected in either group. Overall, stratified analyses indicate that the association between aMED adherence and sleep duration is most evident among racial/ethnic minority adults.

## DISCUSSION

This cross-sectional study examined the relationship between adherence to the aMED and various dimensions of sleep health, with a particular focus on differences by race and ethnicity. Although aMED adherence was not significantly associated with the MDSH score, it was associated with achieving recommended sleep duration. Specifically, stratified analyses revealed that racial/ethnic minority adults with moderate/high adherence to aMED had significantly higher odds of achieving the recommended sleep duration, whereas the association among NH White adults was weaker and not statistically significant. Contrary to study hypotheses, adherence to aMED was not significantly associated with other individual domains, including sleep regularity, satisfaction, alertness, or timing.

Although a statistically significant association between aMED scores and the overall MDSH score was not observed, the point estimates indicated a positive association between higher aMED scores and better sleep health. Most prior work on Mediterranean-style diets has focused on individual sleep outcomes, such as quality, insomnia, and duration,^77,78^ with reviews reporting consistent benefits for duration.^79^ Far fewer studies have examined multidimensional frameworks, such as RU SATED (a self-report questionnaire that assesses sleep health across the following 6 core dimensions: regularity, usage/satisfaction, sleep timing, adequate duration, time in bed/efficiency, and excessive daytime dozing/alertness)^80^ or the MDSH, which limits direct comparisons. Using the MDSH, this study highlights that diet—sleep associations may be domain-specific, with more potent associations with sleep duration than with broader MDSH. In contrast, the null findings for sleep quality, timing, and alertness are consistent with prior studies reporting mixed or nonsignificant associations for these dimensions.^77,79,81^ This suggests that adherence to the alternate Mediterranean diet may be most robustly linked to sleep duration, with more variable effects on other aspects of sleep health. In addition, the authors examined racial/ethnic differences in these associations using a large, diverse US sample. Thus, this study complements existing reviews by demonstrating both the importance of examining multiple sleep dimensions and the potential for contextual variation across population subgroups.

The present findings are consistent with biological pathways identified in prior work. Nutrients central to the Mediterranean diet, such as tryptophan, magnesium, and B vitamins, facilitate the synthesis of serotonin and melatonin, thereby influencing sleep onset.^41,42^ Similarly, n-3 fatty acids and unsaturated fats support neuronal function and slow-wave sleep, while higher saturated fat intake has been linked to less favorable sleep architecture.^41^ The anti-inflammatory and antioxidant profiles of plant-based foods and olive oil may also attenuate systemic inflammation, a pathway implicated in sleep disturbances.^41,42^ Together, these mechanisms suggest that diet quality may exert direct influences on sleep regulation beyond its role in a generally healthy lifestyle, which helps explain the present findings, which revealed stronger associations with sleep duration.

When considering differences by race and ethnicity, stratified analyses provided a more precise interpretation than the interaction models, which rely on a single reference group and can be challenging to interpret. The stratified findings suggest that the association between adherence to a Mediterranean diet and achieving recommended sleep duration is especially evident among racial/ethnic minority adults, whereas no significant association was observed among NH White adults. This pattern is consistent with prior work showing that associations between sleep and diet quality may be stronger among Black and Latino/a and Hispanic adults than among NH White adults.^82^ These associations may also vary depending on broader contextual influences, including food insecurity, socioeconomic stressors, irregular work schedules, and environmental exposures.^83^ Prior research highlights that acculturation,^84^ food security,^85^ and structural inequalities^47,86,87^ can modify behavioral—health relationships, and our study’s findings align with this broader literature. In addition, it is crucial to recognize that the aMED score reflects adherence to an alternate Mediterranean-style dietary pattern, which may not fully capture culturally specific foods and healthy dietary practices across all racial/ethnic groups. Although sex may further modify diet—sleep associations, we did not test these interactions, as doing so would extend beyond the prespecified aims and available statistical power. Taken together, these findings underscore the importance of examining both dietary and social contexts in influencing sleep health.

### Strengths and Limitations

The strengths of the present study include the use of a large and diverse sample of adults in the United States. Another strength was the ability to examine racial/ethnic differences between NH White adults and racial/ethnic minority groups. In addition, although sleep data were self-reported, we used the MDSH score to capture overall sleep health, an approach less commonly used in the literature, which typically focuses on individual sleep dimensions. A further strength was the use of two 24-hour dietary recalls with associated 2-day dietary weights, which provides a more reliable estimate of dietary intake than reliance on a single recall. This strengthens the assessment of overall diet quality and reduces within-person day-to-day variability.^88^

This study is not without limitations. First, the cross-sectional design prevents establishing temporality or causal inference, and it remains possible that sleep duration influences diet quality rather than the reverse. Second, although sleep was assessed using validated self-report questionnaires, the items captured participants’ usual sleep patterns without reference to a specific timeframe, which may reduce temporal alignment with the 24-hour dietary recall data and contribute to nondifferential misclassification. Third, unmeasured confounding may be present, including the use of sleep medications, underlying medical conditions, or other factors related to both diet and sleep. Fourth, the results may not be generalizable to populations outside the United States. Fifth, NHANES does not collect information on the timing of food intake, and the 2 dietary recalls may occur on any combination of weekday or weekend days. As a result, weekday and weekend variability and broader chrononutrition patterns could not be evaluated and may have influenced the characterization of the diet—sleep relationship. Finally, we did not apply the National Cancer Institute usual-intake method.^58^ This method is optimized for estimating the usual intake of individual nutrients or episodically consumed foods, whereas our exposure, the aMED dietary pattern score, is a composite index derived from multiple dietary components. Because the aMED relies on recall-based intake levels rather than modeled usual intake distributions, long-term diet quality may not be fully captured, and nondifferential misclassification is possible. Although smoking status was included as a covariate, it was categorized into 3 groups (ie, never, former, and current smokers). NHANES contains more detailed measures of smoking behavior (eg, intensity, frequency, and recency); collapsing these into broader categories may have introduced residual confounding. Although NHANES collects limited information on work schedule (Occupation Questionnaire question OCQ670), nearly one-half of the participants had missing responses, and the measure does not fully capture rotating or irregular shifts. For these reasons, shift work was not included as a covariate in the present analysis. Another limitation is that sleep measures were dichotomized into “good” vs “poor” categories to construct the MDSH score. Although this approach may reduce nuance and mask gradations within each sleep dimension, it is consistent with the MDSH framework proposed by Buysse^43^ and applied in several large epidemiologic studies.^43,62^ Dichotomization facilitates comparability across sleep domains and simplifies interpretation for population-level research. Nonetheless, future work using continuous measures, alternative cutoffs, or weighted composite scores may capture greater variability and provide a more nuanced understanding of the diet—sleep relationship. Although our study categorized sleep duration as recommended vs nonrecommended according to the National Sleep Foundation guidelines,^59^ we did not further stratify nonrecommended sleep into short vs long sleep. Long sleep duration was relatively uncommon in this sample, which limited the statistical power for subgroup analyses. Future studies with larger samples should examine whether associations differ between short and long sleep or when using continuous measures of sleep duration. Finally, in this study, sleep satisfaction was inferred from whether participants had ever reported sleep problems to a physician or health care professional, as indicated by an NHANES survey item. This measure primarily captures whether someone sought medical attention for sleep issues, such as insomnia, but does not fully encompass the broader concept of sleep satisfaction. Participants may have experienced sleep disturbances without disclosing them to a provider, either due to personal reasons or limited access to health care. Although pragmatic, this proxy measure may oversimplify the construct of sleep satisfaction and introduce nondifferential misclassification, potentially obscuring meaningful differences across racial/ethnic groups and contributing to the lack of observed interaction.

## CONCLUSIONS

In this cross-sectional analysis, adherence to the aMED was not associated with the overall MDSH composite score. However, higher aMED scores were associated with greater odds of achieving recommended sleep duration. This association was observed among racial/ethnic minority adults, but not NH White adults. Given the cross-sectional design, temporality cannot be inferred. Future longitudinal studies incorporating objective sleep measures are needed to clarify the direction and mechanisms underlying these associations and to better understand potential heterogeneity across racial/ethnic groups.

## Figures and Tables

**Table 1.** Sociodemographic, behavioral, dietary, and sleep characteristics of US adults in the 2017-2018 National Health and Nutrition Examination Survey, stratified by adherence to the aMED^a^

		aMED adherence		
Characteristic	Overall sample (n = 3005)	Low (n = 1215)	Moderate/high (n = 1790)	*P* value

	*mean (SE)* ^b^			
**aMED continous score**	4.0 (0.09)	2.3 (0.03)	5.1 (0.04)	<.0001
**Age, y**	47.1 (0.6)	44.8 (0.8)	48.7 (0.8)	.001
	*n (%)* ^c^			
**Sex**				.19
Male	1538 (51.3)	646 (53.3)	892 (49.9)	
Female	1467 (48.8)	569 (46.7)	898 (50.1)	
**Race/ethnicity**				<.0001
Non-Hispanic White	1105 (64.2)	513 (65.8)	592 (63.1)	
Latino/Hispanic	633 (15.0)	214 (13.1)	419 (16.3)	
Non-Hispanic Black	705 (10.6)	332 (12.9)	373 (9.0)	
Non-Hispanic Asian	403 (5.9)	75 (3.3)	328 (7.7)	
Other/multiracial	159 (4.2)	81 (4.9)	78 (3.8)	
**Smoking status**				<.0001
Never	1729 (59.3)	608 (52.4)	1121 (63.9)	
Current	543 (16.2)	313 (22.3)	230 (12.1)	
Former	733 (24.5)	294 (25.3)	439 (24.1)	
**Educational attainment**				<.0001
<9 y	179 (2.5)	65 (2.3)	114 (2.6)	
9-11 y	286 (5.9)	157 (8.9)	129 (3.8)	
12 y	689 (26.3)	353 (34.9)	336 (20.5)	
>12 y	1851 (65.3)	640 (53.9)	1211 (73.0)	
**Marital status**				.05
Never married	593 (20.9)	312 (24.8)	281 (18.3)	
Married/living with partner	1794 (62.9)	648 (58.3)	1146 (66.0)	
Divorced/widowed/separated	618 (16.2)	255 (16.9)	363 (15.7)	
**Poverty-to-income ratio**				<.0001
≥1.0	2246 (81.4)	856 (77.6)	1390 (84.0)	
<1.0	759 (18.6)	359 (22.4)	400 (16.0)	
**Household food security**				<.0001
Full food security	2415 (84.1)	906 (79.0)	1509 (87.5)	
Marginal food security	258 (6.8)	123 (8.4)	135 (5.7)	
Low food security	220 (6.1)	113 (7.1)	107 (5.4)	
Very low food security	112 (3.1)	73 (5.4)	39 (1.5)	
	*mean (SE)* ^b^			
**Body mass index**	29.6 (0.3)	30.5 (0.4)	28.9 (0.4)	.009
**Sedentary behavior, min/wk**	383 (14)	353 (14)	419 (56)	.045
**Moderate to vigorous physical activity, min/wk**	1320 (112)	1591 (143)	1052 (81)	.002
**Caffeine intake, mg/d**	170.7 (4.9)	181.5 (6.5)	163.4 (6.2)	.02
**Daily energy intake, kcal/d**	2110 (24)	1990 (33)	2192 (27)	<.0001
	*n (%)* ^c^			
**Multidimensional sleep health**				.29
Poor	974 (29.2)	438 (31.5)	536 (27.6)	
Good	2031 (70.8)	777 (68.5)	1254 (72.4)	

a aMED =alternate Mediterranean diet.

b Values are presented as weighted mean (SE) for continuous variables.

c Values are presented as unweighted sample sizes (n) with weighted percentages (%) for categorical variables. Percentages may not total 100 due to rounding.

**Table 2.** Association between continuous aMED^a^ score and sleep health outcomes among US adults, 2017-2018 National Health and Nutrition Examination Survey (n = 3,005)

Sleep health outcomes	Model 1^b^		Model 2^c^		Model 3^d^	
	OR^e^ (95% CI)	*P* value	OR (95% CI)	*P* value	OR (95% CI)	*P* value

Good sleep health (MDSH^f^)	1.1 (0.9-1.2)	.17	1.1 (0.9-1.2)	.15	1.0 (0.9-1.2)	.32
Good sleep duration^g^	1.0 (1.0-1.2)	.03	1.1 (1.0-1.2)	.005	1.1 (1.0-1.2)	.03
Good sleep regularity^h^	1.0 (0.9-1.2)	.44	1.0 (0.9-1.1)	.68	1.0 (0.9-1.1)	.93
Good sleep satisfaction^i^	1.0 (0.9-1.1)	.17	1.1 (1.0-1.2)	.03	1.0 (0.9-1.1)	.65
Good sleep alertness^j^	1.0 (0.9-1.1)	.92	1.0 (0.9-1.1)	.97	1.0 (0.9-1.1)	.44
Good sleep timing^k^	1.0 (0.9-1.1)	.50	0.9 (0.8-1.0)	.28	1.0 (0.9-1.1)	.59

a aMED = alternate Mediterranean diet.

b Model 1: Unadjusted model with aMED modeled continuously; OR reflects increased odds per 1-point increase.

c Model 2: Adjusted for energy intake, race, age, and sex; with aMED modeled continuously; OR reflects increased odds per 1-point increase.

d Model 3: Adjusted for energy intake, race, age, sex, smoking history, education level, body mass index, caffeine intake, household food security, and poverty-to-income ratio; with aMED modeled continuously; OR reflects increased odds per 1-point increase.

e OR = odds ratio.

f MDSH = Multidimensional sleep health score; sum of the number of good sleep indicators across 5 domains (ie, timing, regularity, duration, satisfaction, and alertness); range, 0-5; score ≥3 classified as good sleep health.

g Good sleep duration: 7-9 h/night for adults aged 18-64 y; 7-8 h/night for adults 65 y and older.

h Good sleep regularity: Difference in weekday vs weekend sleep midpoint ≤2 h.

i Good sleep satisfaction: Participants reporting no physician-diagnosed sleep troubles were classified as good.

j Good sleep alertness: Participants feeling overly sleepy were never/rarely/sometimes classified as good.

k Good sleep timing: Sleep midpoint between 2:00 and 4:00 am was classified as good.

**Table 3.** Interaction between race/ethnicity and aMED^a^ adherence on odds of sleep health outcomes among US adults, 2017-2018 National Health and Nutrition Examination Survey (n = 3005)

Sleep health outcomes	Group^b^	OR^c^ (95% CI)	*P* value	*P*_interaction_

Multidimensional sleep health^d^				.57
	Low aMED × NH^e^ White adults	1.2 (0.9-1.6)	.22	
	Moderate/high aMED × NH White adults	1.3 (0.7-2.2)	.39	
	Moderate/high aMED × racial/ethnic minority adults	1.2 (0.9-1.6)	.22	
Good sleep duration^f^				.04
	Low aMED × NH White adults	1.4 (0.9-2.0)	.09	
	Moderate/high aMED × NH White adults	1.6 (1.0-2.5)	.04	
	Moderate/high aMED × racial/ethnic minority adults	1.4 (1.1-1.7)	.01	
Good sleep regularity^g^				.28
	Low aMED × NH White adults	1.5 (0.9-2.4)	.10	
	Moderate/high aMED × NH White adults	1.5 (0.8-2.7)	.12	
	Moderate/high aMED × racial/ethnic minority adults	1.3 (0.8-2.3)	.31	
Good sleep satisfaction^h^				.26
	Low aMED × NH White adults	0.7 (0.4-1.0)	.04	
	Moderate/high aMED × NH White adults	0.7 (0.5-1.2)	.16	
	Moderate/high aMED × racial/ethnic minority adults	0.9 (0.7-1.1)	.27	
Good sleep alertness^i^				.64
	Low aMED × NH White adults	1.1 (0.8-1.7)	.53	
	Moderate/high aMED × NH White adults	1.2 (0.7-1.9)	.48	
	Moderate/high aMED × racial/ethnic minority adults	0.9 (0.6-1.4)	.72	
Good sleep timing^j^				.43
	Low aMED × NH White adults	0.9 (0.5-1.4)	.60	
	Moderate/high aMED × NH White adults	0.6 (0.3-1.1)	.10	
	Moderate/high aMED × racial/ethnic minority adults	0.9 (0.7-1.2)	.41	

a aMED = alternate Mediterranean diet.

b Reference group: racial/ethnic minority adults with low aMED adherence.

c OR = odds ratio from model 3, adjusted for age, sex, race and ethnicity, total dietary energy intake, mean caffeine intake, smoking status, education, household food security, poverty-to-income ratio, and body mass index.

d Composite of 5 domains (ie, sleep duration, timing, regularity, satisfaction, and alertness); score range 0-5; good multidimensional sleep health was defined as ≥3.

e NH = non-Hispanic.

f Good sleep duration: 7-9 h/night for adults aged 18-64 y; 7-8 h/night for adults 65 y and older.

g Good sleep regularity: Difference in weekday vs weekend sleep midpoint ≤2 h.

h Good sleep satisfaction: Participants with no physician-diagnosed sleep troubles were classified as good.

i Good sleep alertness: Feeling overly sleepy was never/rarely/sometimes classified as good.

j Good sleep timing: Sleep midpoint between 2:00 and 4:00 am was classified as good.

**Table 4.** Association between alternate Mediterranean diet adherence and odds of sleep health outcomes, stratified by race/ ethnicity among US adults (post hoc analyses; 2017-2018 National Health and Nutrition Examination Survey) (n = 3005)

Sleep health outcomes	OR^a^ (95% CI)	*P* value

**Multidimensional sleep health** ^b^		
NH^c^ White adults with moderate/high adherence vs low adherence	1.0 (0.5-1.8)	.89
Racial/ethnic minority adults with moderate/high adherence vs low adherence	1.3 (1.0-1.7)	.06
**Good sleep duration** ^d^		
NH White adults with moderate/high adherence vs low adherence	1.1 (0.7-1.9)	.68
Racial/ethnic minority adults with moderate/high adherence vs low adherence	1.3 (1.1-1.7)	.01
**Good sleep regularity** ^e^		
NH White adults with moderate/high adherence vs low adherence	1.0 (0.6-1.9)	.89
Racial/ethnic minority adults with moderate/high adherence vs low adherence	1.5 (0.9-2.4)	.13
**Good sleep satisfaction** ^f^		
NH White adults with moderate/high adherence vs low adherence	1.0 (0.7-1.5)	.93
Racial/ethnic minority adults with moderate/high adherence vs low adherence	0.9 (0.7-1.2)	.57
**Good sleep alertness** ^g^		
NH White adults with moderate/high adherence vs low adherence	0.9 (0.6-1.4)	.54
Racial/ethnic minority adults with moderate/high adherence vs low adherence	1.1 (0.7-1.6)	.70
**Good sleep timing** ^h^		
NH White adults with moderate/high adherence vs low adherence	0.7 (0.4-1.4)	.32
Racial/ethnic minority adults with moderate/high adherence vs low adherence	0.8 (0.6-1.0)	.15

a OR = odds ratio from models adjusted for age, sex, total energy intake, caffeine intake, smoking status, educational attainment, household food security, poverty-to-income ratio, and body mass index.

b Good sleep health: multidimensional sleep health score; sum of the number of good sleep indicators across 5 domains (ie, timing, regularity, duration, satisfaction, and alertness); range, 0-5; ≥3 classified as good sleep health.

c NH = non-Hispanic.

d Good sleep duration: 7-9 h/night for adults aged 18-64 y; 7-8 h/night for adults 65 y and older.

e Good sleep regularity: Difference in weekday vs weekend sleep midpoint ≤2 h.

f Good sleep satisfaction: Participants reporting no physician-diagnosed sleep troubles were classified as good.

g Good sleep alertness: Participants feeling overly sleepy never/rarely/sometimes were classified as good.

h Good sleep timing: Sleep midpoint between 2:00 and 4:00 am was classified as good.

## Data Availability

The data used in this study are publicly available from the National Health and Nutrition Examination Survey and can be accessed through the Centers for Disease Control and Prevention website (https://www.cdc.gov/nchs/nhanes/index.html).

## RESEARCH SNAPSHOT

### Research Question:

Does adherence to the alternate Mediterranean Diet relate to multidimensional sleep health among US adults, and do these associations differ by race/ethnicity?

### Key Findings:

In this cross-sectional study of 3005 adults from 2017–2018 National Health and Nutrition Examination Survey, a higher alternate Mediterranean diet score was associated with greater odds of achieving recommended sleep duration, but not overall multidimensional sleep health. Stratified analyses revealed a significant association between alternate Mediterranean diet adherence and sleep duration among racial/ethnic minority adults, but not among non-Hispanic White adults. No other sleep dimensions showed statistically significant associations.
